# Supplementary material for: A mixed-methods comparison of gender differences in alcohol consumption and drinking characteristics among patients in Moshi, Tanzania
Source: PLOS Glob Public Health. 2023 Oct 24;3(10):e0002009. doi: 10.1371/journal.pgph.0002009 (PMC10597514; doi:10.1371/journal.pgph.0002009)
Supplement: S2 File — (PDF) [file pgph.0002009.s004.pdf]

CRERC 07

KILIMANJARO CHRISTIAN MEDICAL UNIVERSITY COLLEGE

(A Constituent College of Tumaini University Makumira)

P. O. Box 2240, MOSHI, Tanzania.

RESEARCH ETHICAL CLEARANCE CERTIFICATE

No. 2515

Research Proposal No. 1297

Study Title: Differences in alcohol use behaviours between men and women in Moshi: A mixed method study

Study Area : KCMC referral hospital

PIs Name : Blandina Mmbaga

Coinvestigators: Catherine Staton, Joao Ricardo Nickenig Vissoci, Alena Pauley, Ashley Phillips

Institution (s) : Kilimanjaro Christian Medical University College

The Proposal was approved by CRERC on : 16th August, 2021

Duration of Study : One year

From : 16th August, 2021 to 15th August, 2022

PROF. MRAMBA NYINDO
Chair – CRERC

PROF. EPHATA KAAYA
PROVOST - KCMU College
