# Supplementary material for: A mixed-methods comparison of gender differences in alcohol consumption and drinking characteristics among patients in Moshi, Tanzania
Source: PLOS Glob Public Health. 2023 Oct 24;3(10):e0002009. doi: 10.1371/journal.pgph.0002009 (PMC10597514; doi:10.1371/journal.pgph.0002009)
Supplement: S1 Questionnaire — (PDF) [file pgph.0002009.s006.pdf]

Screening Questions

Please complete the screening questions below.

Thank you!

1) Registry ID# __________________________________

2) Did participant consent to data sharing?
   - ◯ Yes
   - ◯ No

3) Patient Initials __________________________________

4) Age __________________________________

5) Patient Sex
   - ◯ Male
   - ◯ Female
   - ◯ Other

6) Date __________________________________

7) Hospital Unit
   - ◯ Emergency Room
   - ◯ Reproductive Health Center

8) Reason For Seeking Care __________________________________

9) Injury Status
   - ◯ Injury
   - ◯ Non-Injury

Demographic Information

Please answer the questions below.

Thank you!

1. Dini yako ni ipi? (Religion?)	◯ None<br>◯ Muslim<br>◯ Christian<br>◯ Other<br>◯ Refused/Don't know
Other Religion?	__________________________________
1a. Dini ina umuhimu gani katika maisha yako? (How important is religion in your life?)	◯ Not important<br>◯ Somewhat unimportant<br>◯ Neutral<br>◯ Somewhat important<br>◯ Very important<br>◯ Refused/Don't know
2. Uko kwenye mahusiano gani ya ndoa? (What is your marital status?)	◯ Ninaishi na mwenza katika ndoa iliyosajiliwa (Living together with a partner in a registered marriage)<br>◯ Ninaishi na mwenza katika ndoa ambayo haijasajiliwa (Living together with a partner but not in a registered marriage)<br>◯ Tumeachana/Tumetengana (Divorced or Separated)<br>◯ Mjane/Mgane (Widowed)<br>◯ Sijawahi kuoa/kuolewa (Never Married)<br>◯ Refused/Don't know
3. Mama yako amefikia kiwango gani cha elimu? (What level of education did your mother complete?)	◯ Hakuna kabisa (None or n/a)<br>◯ Elimu ya msingi kidogo (Some primary education)<br>◯ Amemaliza elimu ya msingi (Finished primary)<br>◯ Elimu ya O-level ya sekondari kidogo (Some O level secondary education)<br>◯ Amemaliza elimu ya O-level ya sekondari (Finished O-level secondary)<br>◯ Elimu ya A-level sekondari kidogo (Some A- level secondary)<br>◯ Amemaliza elimu ya A-level sekondari (Finished A-level secondary)<br>◯ Chuo cha ufundi (eg, VETA) (Vocational training centres)<br>◯ Amesoma chuo kidogo (Some college)<br>◯ Amemaliza chuo (Finished college)<br>◯ Amesoma kidogo elimu ya juu baada ya chuo kikuu (Some post college)<br>◯ Amemaliza shahada ya pili au shahada ya tatu (Completed masters or doctoral degree)<br>◯ Refused/Don't know

4. Baba yako amefikia kiwango gani cha elimu? (What level of education did your father complete?)

- ◯ Hakuna kabisa (None orn/a)
- ◯ Elimu ya msingi kidogo (Some primary education)
- ◯ Amemaliza elimu ya msingi (Finished primary)
- ◯ Elimu ya O-level ya sekondari kidogo (Some O level secondary education)
- ◯ Amemaliza elimu ya O-level ya sekondari (Finished O-level secondary)
- ◯ Elimu ya A-level sekondari kidogo (Some A- level secondary)
- ◯ Amemaliza elimu ya A-level sekondari (Finished A-level secondary)
- ◯ Chuo cha ufundi (eg, VETA) (Vocational training centres)
- ◯ Amesoma chuo kidogo (Some college)
- ◯ Amemaliza chuo (Finished college)
- ◯ Amesoma kidogo elimu ya juu baada ya chuo kikuu (Some post college)
- ◯ Amemaliza shahada ya pili au shahada ya tatu (Completed masters or doctoral degree)
- ◯ Refused/Don't Know

5. Umefikia kiwango gani cha elimu? (What level of education did you complete?)

- ◯ Sina elimu yoyote (No education)
- ◯ Elimu ya msingi kidogo (Some primary education)
- ◯ Nimemaliza elimu ya msingi (Finished primary)
- ◯ Elimu ya O-level ya sekondari kidogo (Some O level secondary education)
- ◯ Nimemaliza elimu ya O-level ya sekondari (Finished O-level secondary)
- ◯ Elimu ya A-level sekondari kidogo (Some A- level secondary)
- ◯ Nimemaliza elimu ya A-level sekondari (Finished A-level secondary)
- ◯ Chuo cha ufundi (eg, VETA)(Vocational training centres)
- ◯ Nimesoma chuo kidogo (Some college)
- ◯ Nimemaliza chuo (Finished college)
- ◯ Nimesoma kidogo elimu ya juu baada ya chuo kikuu (Some post college)
- ◯ Nimemaliza shahada ya pili au shahada ya tatu (Completed masters or doctoral degree)
- ◯ Refused/Don't know

6. Years of education?

____

7. Je kwa sasa umeajiriwa au umejiajiri? (Are you currently employed?)

- ◯ Hapana - Ni mwanafunzi (No I am a student)
- ◯ Hapana na si mwanafunzi (No and I am not a student)
- ◯ Ndiyo (Yes)
- ◯ Refused/Don't know

7a. Je, kazi yako ya sasa ni ipi? (What is your current employment?)

- ◯ Mtaalamu (Professional)
- ◯ Karani (Clerical)
- ◯ Mauzo (Sales)
- ◯ Za nyumbani (Household)
- ◯ Kilimo cha kujiajiri (Agricultural - self-employed)
- ◯ Kilimo cha kuajiriwa (Agricultural - employee)
- ◯ Huduma (Services)
- ◯ Kazi za mikono zenye ujuzi (Skilled manual)
- ◯ Kazi za mikono zisizo za ujuzi (Unskilled manual)
- ◯ Other
- ◯ Refused/Don't know

Other employment

____

8. Je! Kabila lako ni lipi?
(What tribe do you affiliate with?)

- ◯ Chagga
- ◯ Pare
- ◯ Sambaa
- ◯ Maasai
- ◯ Iraq
- ◯ Sukuma
- ◯ Mmeru
- ◯ Nyaturu
- ◯ Muha
- ◯ Other African
- ◯ Non-African
- ◯ Refused/Don't know

Which OTHER Tribe?

____

Non-African Race

- ◯ White
- ◯ Asian
- ◯ Black/African American
- ◯ American Indian/Alaska Native
- ◯ Native Hawaiian or Pacific Islander
- ◯ Refused/DK

Non-African Ethnicity

- ◯ Hispanic, Latino, or of Spanish origin
- ◯ NOT Hispanic, Latino, or of Spanish origin
- ◯ Refused/Don't know

9. Watu wanaoishi kwenye kaya yako (pamoja na wewe)?
(Number of People Living in your household, including you?)

____

10. Kipato chako ni (kwa mwezi)?
(What is your personal income, per month?)

____

Personal Income (Categorical)

- ◯ 0 to 50000TZS
- ◯ >50000 to 100000 TZS
- ◯ >100000 to 150000TZS
- ◯ >150000 to 200000
- ◯ >200000

11. JUMLA ya mapato kwenye kaya yenu ni (kwa mwezi)?
(What is your TOTAL HOUSEHOLD income, per month?)

____

Total Household Income (Categorical)

- ◯ 0 to 50000TZS
- ◯ >50000 to 100000 TZS
- ◯ >100000 to 150000TZS
- ◯ >150000 to 200000
- ◯ >200000

12. Inachukua muda gani kufika kwenye kituo cha afya cha karibu yako (in hours)?
(How many hours does it take to get to your nearest health facility?)

____

13. Inachukua muda gani wewe kufika KCMC (in hours)?
(How many hours does it take you to get to KCMC?)

____

Alcohol-Related Demographic Information

Please answer the questions below.

Thank you!

Question	Response
1. Kama ungekunywa pombe, ni aina gani ungependelea? (If you were to drink alcohol, which type would you prefer?)	◯ Wine<br>◯ Light Beer<br>◯ Beer<br>◯ Liquor/Spirits<br>◯ Dadii<br>◯ Mbege<br>◯ Ulanzi<br>◯ Piwa<br>◯ Gongo<br>◯ Changaa<br>◯ None<br>◯ Other<br>◯ Refused/Don't know
Which OTHER type of alcohol?	__________________________________
1a. Ni mara ngapi unakunywa pombe? (How often do you drink?)	◯ 0 times per week (0 kwa wiki)<br>◯ 1 to 2 times per week (Mara 2 kwa wiki)<br>◯ 3 to 4 times per week Mara (3-4 kwa wiki)<br>◯ 5 to 6 times per week (5-6 chupa)<br>◯ Every day (Kila siku)<br>◯ Multiple times Per day (Mara nyingi kwsiku)<br>◯ Refused/Don't Know
1b. Unakunywa kiasi gani cha pombe? (When you drink, how much do you drink?)	◯ 0 chupa (0 drinks)<br>◯ 1-2 chupa (1 to 2 bottles)<br>◯ 3-4 chupa (3 to 4 drinks)<br>◯ 5-6 chupa (5 to 6 bottles)<br>◯ >6 chupa 6 zaidi (>6 bottles)<br>◯ Refused/Don't Know
2. Unatumia kiasi gani cha pesa kwenye pombe kwa wiki?<br><br>(How much do you spend on alcohol (per week)?	◯ 0 to 10000 TZS<br>◯ 10000 to 50000 TZS<br>◯ 50000 TZS to 100000 TZS<br>◯ >100000 TZS<br>◯ Refused/Don't Know
How much alcohol is safe to drink while pregnant?	◯ 0 chupa (0 drinks)<br>◯ 1-2 chupa/wiki (1 to 2 bottles per week)<br>◯ 3-4 chupa/wiki (3 to 4 bottles per week)<br>◯ 5-6 chupa/wiki (5 to 6 bottles per week)<br>◯ 7-9 chupa/wiki (7 to 9 bottles per week)<br>◯ ≥10 chupa (≥10 bottles per week)<br>◯ Refused/Don't Know
During a pregnancy, when is it okay for a woman to drink?	◯ It is never okay<br>◯ Beginning of pregnancy<br>◯ Middle of pregnancy<br>◯ End of Pregnancy<br>◯ It is okay at any time<br>◯ Refused/Don't Know

3. Je, wewe ni mjamzito? (Are you pregnant?)	◯ Yes<br>◯ No<br>◯ n/a (patient is a male)<br>◯ Refused/Don't know
3a. Je, umetumia pombe tangu upate ujauzito?<br>(Have you consumed alcohol since becoming pregnant?)	◯ Yes<br>◯ No<br>◯ Refused/Don't know
3b. Je, tabia yako ya unywaji imebadilika tangu upate ujauzito?<br>(Has your drinking behavior changed since becoming pregnant?)	◯ Ndio, Nimetumia pombe nyingi zaidi tangu niwe mjamzito (Yes, I have consumed more alcohol since becoming pregnant)<br>◯ Ndio, Nimetumia pombe kidogo zaidi tangu niwe mjamzito (Yes, I have consumed less alcohol since becoming pregnant)<br>◯ Hapana, Nimetumia kiwango kile kile cha pombe kama nililchotumia kabla sijawa mjamzito (No, I have consumed the same amount of alcohol now as I did before becoming pregnant)<br>◯ Refused/Dont't Know
4. Je, umewahi kuwa mjauzito kabla?<br>(Have you ever been pregnant before?)	◯ Yes<br>◯ No<br>◯ n/a (patient is a male)<br>◯ Refused/Don't know
4a. Je, umewahi kutumia pombe ukiwa mjamzito?<br>(Have you ever consumed alcohol while pregnant?)	◯ Yes<br>◯ No<br>◯ Refused/Don't know
4b. Je, tabia yako unywaji ilibadilikaje ulipokuwa mjamzito?<br>(How would your drinking behavior change when you were pregnant?)	◯ Nilitumia pombe nyingi zaidi nikiwa mjamzito (I consumed more alcohol while being pregnant)<br>◯ Nilitumia pombe kidogo zaidi nikiwa mjamzito (I consumed less alcohol while being pregnant)<br>◯ Nilitumia kiwango kile kile cha pombe nikiwa mjamzito kama kabla sijawa mjamzito (I consumed the same amount of alcohol while pregnant as while not pregnant)<br>◯ Refused/Dont't Know
4c. How much alcohol did you consume while pregnant?	◯ 0 chupa (0 drinks)<br>◯ 1-2 chupa/wiki (1 to 2 bottles per week)<br>◯ 3-4 chupa/wiki (3 to 4 bottles per week)<br>◯ 5-6 chupa/wiki (5 to 6 bottles per week)<br>◯ 7-9 chupa/wiki (7 to 9 bottles per week)<br>◯ ≥10 chupa (≥10 bottles per week)<br>◯ Refused/Don't Know
5. Wanaume wangapi wanaishi kwenye kaya yako? (kutoa wewe)<br>(How many males live in your household, not including you?)	________________________
6. Kuna mwanaume yoyote kwenye kaya yako anayekunywa pombe?<br>(Do any of the males in your household drink?)	◯ Yes<br>◯ No<br>◯ Refused/Don't know
6a. For the male who drinks the most in your household, how many bottles does he drink in a week?	________________________

6b. For the male who drinks the second most in your household, how many bottles does he drink in a week?	__________________
7. Wanawake wangapi wanaishi kwenye kaya yako? (kutoa wewe)<br>(How many females live in your household, not including you?)	__________________
8. Kuna mwanamke yoyote kwenye kaya yako anayekunywa pombe?<br>(Do any of the females in your household drink?)	◯ Yes<br>◯ No<br>◯ Refused/Don't know
8a. For the female who drinks the most in your household, how many bottles does she drink in a week?	__________________
8b. For the female who drinks the second most in your household, how many bottles does she drink in a week?	__________________
9. Je, umewahi kujaribu kuacha kunywa pombe?<br>(Have you ever wanted to quit drinking alcohol?)	◯ Yes<br>◯ No<br>◯ Refused/Don't know
9a. Kwanini ulitamani kuacha? (Why have you wanted to quit?)	◯ Personal reasons (Sababu binafsi)<br>◯ Financial reasons (Sababu za kifedha)<br>◯ Spiritual reasons (Sababu za kiroho)<br>◯ Family reasons (Sababu za kifamilia)<br>◯ Health reasons (Sababu za kiafya)<br>◯ Other (Sababu nyingine)<br>◯ Refused/Don't Know
Kama ipo, itaje (If other, please specify)	__________________
10. Je, unafikiria matumizi ya pombe kupindukia ni tatizo la kiafya/ kitabibu (Do you consider unhealthy alcohol use to be a medical condition?)	◯ Yes<br>◯ No<br>◯ Refused/Don't know
11. Je, umeshawahi kutafuta matibabu kwa ajili ya matumizi ya pombe?<br>(Have you ever sought treatment for alcohol use?)	◯ Yes<br>◯ No<br>◯ Refused/Don't know
12. Je, umeshawahi kutafuta matibabu kwa ajili ya ugonjwa wa akili?<br>(Have you ever sought treatment for psychiatric disease?)	◯ Yes<br>◯ No<br>◯ Refused/Don't know
13. Je, kuna mwanafamilia ambaye ameshawahi kutafuta matibabu kwa ajili ya matumizi ya pombe?<br>(Has a family member ever sought treatment for their alcohol use?)	◯ Yes<br>◯ No<br>◯ Refused/Don't know
14. Je, kuna mwanafamilia ambaye ameshawahi kutafuta matibabu kwa ajili ya ugonjwa wa akili?<br>(Has a family member ever sought treatment for psychiatric disease?)	◯ Yes<br>◯ No<br>◯ Refused/Don't know

AUDIT

Please complete the AUDIT questionnaire below.

Thank you!

1. Kwa mwaka uliopita ni mara ngapi unatumia kinywaji kilicho na kilevi?<br>(How often during the last year do you have a drink containing alcohol?)	◯ Hakuna (Never)<br>◯ Kila mwezi au chini ya mwezi (Monthly or less)<br>◯ Mara 2 hadi 4 /mwezi (2 to 4 times a month)<br>◯ Mara 2 hadi 3 /wiki (2 to 3 times a week)<br>◯ 4 au zaidi / wiki (4 or more times a week)
2. Kwa mwaka uliopita kwa siku ya kawaidia unatumia vinywaji vingapi vyenye kilevi unapokuwa unakunywa?<br>(How many drinks containing alcohol do you have on a typical day when you are drinking during the last year?)	◯ 1 or 2<br>◯ 3 or 4<br>◯ 5 or 6<br>◯ 7 to 9<br>◯ 10 or more
3. Kwa mwaka uliopita ni mara ngapi unatumia vinywaji sita au zaidi kwa mara moja kwa mwaka uliopita?<br>(How often during the last year do you have six or more drinks on one occasion?)	◯ Haijawahi kutokea (Never)<br>◯ Chini ya kila mwezi (Less than monthly)<br>◯ Kila Mwezi (Monthly)<br>◯ Kwa wiki (Weekly)<br>◯ Kila siku au karibu kila siku (Daily/ almost daily)
4. Mara ngapi katika mwaka uliopita uligundua hukuweza kuacha kunywa mara ukishaanza?<br>(How often during the last year have you found that you were not able to stop drinking once you had started?)	◯ Haijawahi kutokea (Never)<br>◯ Chini ya kila mwezi (Less than monthly)<br>◯ Kila Mwezi (Monthly)<br>◯ Kwa wiki (Weekly)<br>◯ Kila siku au karibu kila siku (Daily/ almost daily)
5. Mara ngapi katika mwaka uliopita ulishindwa kufanya unavyotarajiwa kutoka kwako kwa sababu ya kunywa? (How often during the last year have you failed to do what was normally expected of you because of drinking?)	◯ Haijawahi kutokea (Never)<br>◯ Chini ya kila mwezi (Less than monthly)<br>◯ Kila Mwezi (Monthly)<br>◯ Kwa wiki (Weekly)<br>◯ Kila siku au karibu kila siku (Daily/ almost daily)
6. Mara ngapi katika mwaka uliopita ulihitaji kinywaji cha kwanza asubuhi ili kuweza kuendelea na shughuli zako baada ya kunywa sana?<br>(How often during the last year have you needed a first drink in the morning to get yourself going after a heavy drinking session?)	◯ Haijawahi kutokea (Never)<br>◯ Chini ya kila mwezi (Less than monthly)<br>◯ Kila Mwezi (Monthly)<br>◯ Kwa wiki (Weekly)<br>◯ Kila siku au karibu kila siku (Daily/ almost daily)
7. Mara ngapi katika mwaka uliopita ulijihisi kuwa na hatia au kujilaumu baada ya kunywa?<br>(How often during the last year have you had a feeling of guilt or remorse after drinking?)	◯ Haijawahi kutokea (Never)<br>◯ Chini ya kila mwezi (Less than monthly)<br>◯ Kila Mwezi (Monthly)<br>◯ Kwa wiki (Weekly)<br>◯ Kila siku au karibu kila siku (Daily/ almost daily)
8. Mara ngapi katika mwaka uliopita hukuweza kukumbuka kilichotendeka usiku uliopita kwa sababu ulikunywa?<br>(How often during the last year have you been unable to remember what happened the night before because of your drinking?)	◯ Haijawahi kutokea (Never)<br>◯ Chini ya kila mwezi (Less than monthly)<br>◯ Kila Mwezi (Monthly)<br>◯ Kwa wiki (Weekly)<br>◯ Kila siku au karibu kila siku (Daily/ almost daily)
9. Je, umejeruhiwa au mtu mwingine kujeruhiwa kwa sababu ya kunywa kwako?<br>(Have you or someone else been injured because of your drinking?)	◯ Hapana (No)<br>◯ Ndiyo, lakini si kwa mwaka uliopita (Yes, but not in the last year)<br>◯ Ndiyo kwa mwaka uliopita (Yes, during the last year)

9c. Je, unywaji wako wa pombe umewahi kusababisha wewe au mtu mwingine kuumia? (Have you or someone else been injured because of your drinking?)	◯ Hapana (No)<br>◯ Ndiyo, lakini si kwa mwaka uliopita (Yes, but not in the last year)<br>◯ Ndiyo kwa mwaka uliopita (Yes, during the last year)
10. Je, ndugu yako au rafiki yako au daktari au mhudumu wa afya mwingine ameguswa na kunywa kwako au kupendekeza upunguze kunywa kwako?<br>(Has a relative, friend, doctor, or other health care worker been concerned about your drinking or suggested you cut down?)	◯ Hapana (No)<br>◯ Ndiyo, lakini si kwa mwaka uliopita (Yes, but not in the last year)<br>◯ Ndiyo kwa mwaka uliopita (Yes, during the last year)

DrInC

Please complete the DrInC survey below.

Thank you!

E1. Nilikuwa na uchovu wa ulevi/Hangover au Nilihisi vibaya baada ya kunywa.	◯ Haijawahi kutokea (Never)<br>◯ Mara 1 au mara chache (Once or a few times)<br>◯ Mara 1 au 2 kwa wiki (Once or twice a week)<br>◯ Kila siku au karibu kila siku (Daily or almost daily)<br>(MAELEKEZO: Haya ni baadhi ya matukio ambayo wanywaji huwa wanayapitia wakati mwingine. Nitasoma kila moja kwa makini. Tafadhali onyesha ni mara ngapi kila moja lilikutokea. KATIKA MIEZI 3 ILIYOPITA, Hii ilikutokea kama mara ngapi?)
E2. Nilijichukia vibaya mimi mwenyewe kwa sababu ya unywaji wangu.	◯ Haijawahi kutokea<br>◯ Mara 1 au mara chache<br>◯ Mara 1 au 2 kwa wiki<br>◯ Kila siku au karibu kila siku<br>(KATIKA MIEZI 3 ILIYOPITA, Hii ilikutokea kama mara ngapi?)
E3. Kuna siku nimekosa kwenda kazini au shuleni kwa sababu ya unywaji wangu.	◯ Haijawahi kutokea<br>◯ Mara 1 au mara chache<br>◯ Mara 1 au 2 kwa wiki<br>◯ Kila siku au karibu kila siku<br>(KATIKA MIEZI 3 ILIYOPITA, Hii ilikutokea kama mara ngapi?)
E4. Familia yangu au marafiki zangu wamekuwa na wasiwasi au manungu'uniko kuhusu unywaji wangu.	◯ Haijawahi kutokea<br>◯ Mara 1 au mara chache<br>◯ Mara 1 au 2 kwa wiki<br>◯ Kila siku au karibu kila siku<br>(KATIKA MIEZI 3 ILIYOPITA, Hii ilikutokea kama mara ngapi?)
E5. Ninafurahia radha ya bia, mvinyo au pombe kali.	◯ Haijawahi kutokea<br>◯ Mara 1 au mara chache<br>◯ Mara 1 au 2 kwa wiki<br>◯ Kila siku au karibu kila siku<br>(KATIKA MIEZI 3 ILIYOPITA, Hii ilikutokea kama mara ngapi?)
E6. Ubora wa kazi yangu umetetereka kwa sababu ya unywaji wangu.	◯ Haijawahi kutokea<br>◯ Mara 1 au mara chache<br>◯ Mara 1 au 2 kwa wiki<br>◯ Kila siku au karibu kila siku<br>(KATIKA MIEZI 3 ILIYOPITA, Hii ilikutokea kama mara ngapi?)
E7. Uwezo wangu wa kuwa mzazi bora umeathiriwa na unywaji wangu.	◯ Haijawahi kutokea<br>◯ Mara 1 au mara chache<br>◯ Mara 1 au 2 kwa wiki<br>◯ Kila siku au karibu kila siku

E8. Baada ya kunywa, nimepata shida kulala, kusinzia au jinamizi.	◯ Haijawahi kutokea<br>◯ Mara 1 au mara chache<br>◯ Mara 1 au 2 kwa wiki<br>◯ Kila siku au karibu kila siku
E9. Nimeendesha gari baada ya kupata vinywaji vitatu au zaidi.	◯ Haijawahi kutokea<br>◯ Mara 1 au mara chache<br>◯ Mara 1 au 2 kwa wiki<br>◯ Kila siku au karibu kila siku
E10. Unywaji wangu umenisababishia kutumia madawa mengine zaidi ya kulevya.	◯ Haijawahi kutokea<br>◯ Mara 1 au mara chache<br>◯ Mara 1 au 2 kwa wiki<br>◯ Kila siku au karibu kila siku
E11. Nimekuwa mgonjwa na kutapika baada ya kunywa.	◯ Haijawahi kutokea<br>◯ Mara 1 au mara chache<br>◯ Mara 1 au 2 kwa wiki<br>◯ Kila siku au karibu kila siku
E12. Nimekuwa sina furaha kwa sababu ya kunywa kwangu.	◯ Haijawahi kutokea<br>◯ Mara 1 au mara chache<br>◯ Mara 1 au 2 kwa wiki<br>◯ Kila siku au karibu kila siku
E13. Kwa sababu ya kunywa kwangu, nimekuwa sili vizuri.	◯ Haijawahi kutokea<br>◯ Mara 1 au mara chache<br>◯ Mara 1 au 2 kwa wiki<br>◯ Kila siku au karibu kila siku
E14. Nimeshindwa kufanya kilichotarajiwa kwangu kwa sababu ya unywaji wangu.	◯ Haijawahi kutokea<br>◯ Mara 1 au mara chache<br>◯ Mara 1 au 2 kwa wiki<br>◯ Kila siku au karibu kila siku
E15. Unywaji umenisaidia mimi kupumzika	◯ Haijawahi kutokea<br>◯ Mara 1 au mara chache<br>◯ Mara 1 au 2 kwa wiki<br>◯ Kila siku au karibu kila siku
E16. Nimehisi mwenye hatia au kujiaibisha kwa sababu ya unywaji wangu.	◯ Haijawahi kutokea<br>◯ Mara 1 au mara chache<br>◯ Mara 1 au 2 kwa wiki<br>◯ Kila siku au karibu kila siku
E17. Wakati nakunywa, nimesema au kufanya vitu vya kuaibisha.	◯ Haijawahi kutokea<br>◯ Mara 1 au mara chache<br>◯ Mara 1 au 2 kwa wiki<br>◯ Kila siku au karibu kila siku
E18. Wakati nakunywa, utu wangu umebadilika vibaya sana.	◯ Haijawahi kutokea<br>◯ Mara 1 au mara chache<br>◯ Mara 1 au 2 kwa wiki<br>◯ Kila siku au karibu kila siku

E19. Nimejiweka kwenye hatari za kijinga wakati nikiwa nimekunywa.	◯ Haijawahi kutokea<br>◯ Mara 1 au mara chache<br>◯ Mara 1 au 2 kwa wiki<br>◯ Kila siku au karibu kila siku
E20. Nimejikuta kwenye matatizo kwa sababu ya unywaji.	◯ Haijawahi kutokea<br>◯ Mara 1 au mara chache<br>◯ Mara 1 au 2 kwa wiki<br>◯ Kila siku au karibu kila siku
E21. Wakati nakunywa au kutumia madawa ya kulevya, niliongea kwa ukali au kufanya ukatili kwa mtu fulani.	◯ Haijawahi kutokea<br>◯ Mara 1 au mara chache<br>◯ Mara 1 au 2 kwa wiki<br>◯ Kila siku au karibu kila siku
E22. Wakati nakunywa, nimefanya vitu kwa maamuzi ya ghafla ambavyo nilivijutia baadae	◯ Haijawahi kutokea<br>◯ Mara 1 au mara chache<br>◯ Mara 1 au 2 kwa wiki<br>◯ Kila siku au karibu kila siku
E23. Nimejikuta nikipigana wakati nakunywa	◯ Haijawahi kutokea<br>◯ Mara 1 au mara chache<br>◯ Mara 1 au 2 kwa wiki<br>◯ Kila siku au karibu kila siku
E24. Afya yangu kimwili imeathiriwa na unywaji wangu.	◯ Haijawahi kutokea<br>◯ Mara 1 au mara chache<br>◯ Mara 1 au 2 kwa wiki<br>◯ Kila siku au karibu kila siku
E25. Unywaji umenisaidia kuwa na mtazamo chanya zaidi juu ya maisha.	◯ Haijawahi kutokea<br>◯ Mara 1 au mara chache<br>◯ Mara 1 au 2 kwa wiki<br>◯ Kila siku au karibu kila siku
E26. Nimekuwa na matatizo ya kifedha kwa sababu ya unywaji wangu.	◯ Haijawahi kutokea<br>◯ Mara 1 au mara chache<br>◯ Mara 1 au 2 kwa wiki<br>◯ Kila siku au karibu kila siku
E27. Ndoa yangu au mahusiano yangu ya kimapenzi yameathiriwa na unywaji wangu wa pombe.	◯ Haijawahi kutokea<br>◯ Mara 1 au mara chache<br>◯ Mara 1 au 2 kwa wiki<br>◯ Kila siku au karibu kila siku
E28. Nimevuta tumbaku zaidi wakati nikiwa nakunywa.	◯ Haijawahi kutokea<br>◯ Mara 1 au mara chache<br>◯ Mara 1 au 2 kwa wiki<br>◯ Kila siku au karibu kila siku
E29. Muonekano wangu wa kimwili umeathiriwa na unywaji wangu.	◯ Haijawahi kutokea<br>◯ Mara 1 au mara chache<br>◯ Mara 1 au 2 kwa wiki<br>◯ Kila siku au karibu kila siku

E30. Familia yangu imeumizwa na unywaji wangu	◯ Haijawahi kutokea<br>◯ Mara 1 au mara chache<br>◯ Mara 1 au 2 kwa wiki<br>◯ Kila siku au karibu kila siku
E31. Urafiki au mahusiano ya karibu yamekuwa yakiharibiwa na unywaji wangu.	◯ Haijawahi kutokea<br>◯ Mara 1 au mara chache<br>◯ Mara 1 au 2 kwa wiki<br>◯ Kila siku au karibu kila siku
E32. Nimekuwa na uzito wa kupitiliza kwa sababu ya unywaji wangu	◯ Haijawahi kutokea<br>◯ Mara 1 au mara chache<br>◯ Mara 1 au 2 kwa wiki<br>◯ Kila siku au karibu kila siku
E33. Matumizi ya pombe yameathiri uwezo wangu wa kufanya tendo la ndoa.	◯ Haijawahi kutokea<br>◯ Mara 1 au mara chache<br>◯ Mara 1 au 2 kwa wiki<br>◯ Kila siku au karibu kila siku
E34. Nimepoteza hamu ya kufanya shughuli na vitu nivipendayo kwa sababu ya unywaji wangu.	◯ Haijawahi kutokea<br>◯ Mara 1 au mara chache<br>◯ Mara 1 au 2 kwa wiki<br>◯ Kila siku au karibu kila siku
E35. Nikiwa nakunywa, maisha yangu ya kijamii yamekuwa yenye furaha zaidi	◯ Haijawahi kutokea<br>◯ Mara 1 au mara chache<br>◯ Mara 1 au 2 kwa wiki<br>◯ Kila siku au karibu kila siku
E36. Maisha yangu ya kiroho na kimaadili yameathiriwa na unywaji wangu.	◯ Haijawahi kutokea<br>◯ Mara 1 au mara chache<br>◯ Mara 1 au 2 kwa wiki<br>◯ Kila siku au karibu kila siku
E37. Kwa sababu ya unywaji wangu, sijaweza kuwa na aina ya maisha ambayo ninayataka	◯ Haijawahi kutokea<br>◯ Mara 1 au mara chache<br>◯ Mara 1 au 2 kwa wiki<br>◯ Kila siku au karibu kila siku
E38. Unywaji wangu umeniathiri mimi kuwa mtu bora	◯ Haijawahi kutokea<br>◯ Mara 1 au mara chache<br>◯ Mara 1 au 2 kwa wiki<br>◯ Kila siku au karibu kila siku
E39. Unywaji wangu umeharibu maisha yangu ya kijamii, umaarufu au sifa nzuri.	◯ Haijawahi kutokea<br>◯ Mara 1 au mara chache<br>◯ Mara 1 au 2 kwa wiki<br>◯ Kila siku au karibu kila siku
E40. Nimetumia sana au nimepoteza pesa nyingi kwasababu ya unywaji wangu.	◯ Haijawahi kutokea<br>◯ Mara 1 au mara chache<br>◯ Mara 1 au 2 kwa wiki<br>◯ Kila siku au karibu kila siku

E41. Nimekamatwa kwa kuendesha nikiwa na ushawishi wa pombe.	◯ Haijawahi kutokea<br>◯ Mara 1 au mara chache<br>◯ Mara 1 au 2 kwa wiki<br>◯ Kila siku au karibu kila siku
E42. Nimekuwa na matatizo na sheria (Ukiacha kuendesha wakati nimelewa) kwa sababu ya kunywa kwangu.	◯ Haijawahi kutokea<br>◯ Mara 1 au mara chache<br>◯ Mara 1 au 2 kwa wiki<br>◯ Kila siku au karibu kila siku
E43. Nimepoteza ndoa yangu au mahusiano ya karibu ya kimapenzi kwa sababu ya unywaji wangu.	◯ Haijawahi kutokea<br>◯ Mara 1 au mara chache<br>◯ Mara 1 au 2 kwa wiki<br>◯ Kila siku au karibu kila siku
E44. Nimesimamishwa/kufukuzwa au kuacha kazi au shule kwa sababu ya unywaji wangu.	◯ Haijawahi kutokea<br>◯ Mara 1 au mara chache<br>◯ Mara 1 au 2 kwa wiki<br>◯ Kila siku au karibu kila siku
E45. Nilikunywa pombe kawaida, bila matatizo yoyote.	◯ Haijawahi kutokea<br>◯ Mara 1 au mara chache<br>◯ Mara 1 au 2 kwa wiki<br>◯ Kila siku au karibu kila siku
E46. Nimepoteza rafiki kwasababu ya unywaji wangu.	◯ Haijawahi kutokea<br>◯ Mara 1 au mara chache<br>◯ Mara 1 au 2 kwa wiki<br>◯ Kila siku au karibu kila siku
E47. Nimepata ajali nikiwa nimekunywa au nimelewa.	◯ Haijawahi kutokea<br>◯ Mara 1 au mara chache<br>◯ Mara 1 au 2 kwa wiki<br>◯ Kila siku au karibu kila siku
E48. Nikiwa nakunywa au kulewa, nimeumia kimwili, kujeruhiwa au kuungua.	◯ Haijawahi kutokea<br>◯ Mara 1 au mara chache<br>◯ Mara 1 au 2 kwa wiki<br>◯ Kila siku au karibu kila siku
E49. Nikiwa nakunywa au kulewa, nimemjeruhi mtu mwingine.	◯ Haijawahi kutokea<br>◯ Mara 1 au mara chache<br>◯ Mara 1 au 2 kwa wiki<br>◯ Kila siku au karibu kila siku
E50. Nimevunja vitu nikiwa nakunywa au kulewa.	◯ Haijawahi kutokea<br>◯ Mara 1 au mara chache<br>◯ Mara 1 au 2 kwa wiki<br>◯ Kila siku au karibu kila siku

PHQ-9

Please complete the survey below.

Thank you!

123) 1. Katika wiki mbili zilizopita ni kwa siku ngapi umekuwa ukisumbuliwa na kutokuwa na hamu au shauku ya kufanya vitu unavyovipenda?
(In the last two weeks, how often have you been bothered by little interest or pleasure in doing things?)

- ◯ Hakuna kabis (Not at all)
- ◯ Siku kadhaa (Some Days)
- ◯ Zaidi ya nusu ya siku zote (More than half the days)
- ◯ Karibu kila siku (Nearly every day)

124) 2. Katika wiki mbili zilizopita, ni kwa siku ngapi umekuwa ukisumbuliwa na kuzubaa,msongo wa mawazo (stress) au kukosa matumaini?
(In the last two weeks, how often have you been bothered by feeling down, depressed, or hopeless?)

- ◯ Hakuna kabis (Not at all)
- ◯ Siku kadhaa (Some Days)
- ◯ Zaidi ya nusu ya siku zote (More than half the days)
- ◯ Karibu kila siku (Nearly every day)

125) 3.Katika wiki mbili zilizopita, ni kwa siku ngapi umekuwa ukisumbuliwa na kukosa usingizi, kulala bila kushtuka shtuka, au kulala kupita kiasi?
(In the last two weeks, how often have you been bothered by trouble falling or staying asleep, or sleeping too much?)

- ◯ Hakuna kabis (Not at all)
- ◯ Siku kadhaa (Some Days)
- ◯ Zaidi ya nusu ya siku zote (More than half the days)
- ◯ Karibu kila siku (Nearly every day)

126) 4. Katika wiki mbili zilizopita, ni kwa siku ngapi umesumbuliwa na kujisikia umechoka au una nguvu kidogo?
(In the last two weeks, how often have you been bothered by feeling tired or having little energy?)

- ◯ Hakuna kabis (Not at all)
- ◯ Siku kadhaa (Some Days)
- ◯ Zaidi ya nusu ya siku zote (More than half the days)
- ◯ Karibu kila siku (Nearly every day)

127) 5. Katika wiki mbili zilizopita, ni kwa siku ngapi umekuwa ukisumbuliwa na kukosa hamu ya kula au kula zaidi?
(In the last two weeks, how often have you been bothered by poor appetite or overeating?)

- ◯ Hakuna kabis (Not at all)
- ◯ Siku kadhaa (Some Days)
- ◯ Zaidi ya nusu ya siku zote (More than half the days)
- ◯ Karibu kila siku (Nearly every day)

128) 6. Katika wiki mbili zilizopita, ni kwa siku ngapi umekuwa ukisumbuliwa na hisia mbaya kuhusu wewe mwenyewe, au kwamba umeshindwa kufanikiwa, au kuwa umejiangusha au umeiangusha familia yako?
(In the last two weeks, how often have you been bothered by feeling bad about yourself, or that you are a failure, or that you have let yourself or your family down?

- ◯ Hakuna kabis (Not at all)
- ◯ Siku kadhaa (Some Days)
- ◯ Zaidi ya nusu ya siku zote (More than half the days)
- ◯ Karibu kila siku (Nearly every day)

129) 7. Katika wiki mbili zilizopita, ni kwa siku ngapi umesumbuliwa na ugumu wa kuzingatia, kama kuzingatia unachofanya?
(In the last two weeks, how often have you been bothered by trouble concentrating on things, such as what you are doing?)

- ◯ Hakuna kabis (Not at all)
- ◯ Siku kadhaa (Some Days)
- ◯ Zaidi ya nusu ya siku zote (More than half the days)
- ◯ Karibu kila siku (Nearly every day)

130) 8. Katika wiki mbili zilizopita, ni kwa siku ngapi umesumbuliwa na kutembea, kufanya vitendo au kuzungumza polepole sana mpaka watu wengine wangeweza kugundua au kinyume chake, kukosa utulivu au kuhangaika sana kuliko kawaida?
(In the last two weeks, how often have you been bothered by moving or speaking so slowly that other people could have noticed. Or the opposite, being so fidgety or restless that you have been moving around a lot more than usual?)

- ◯ Hakuna kabis (Not at all)
- ◯ Siku kadhaa (Some Days)
- ◯ Zaidi ya nusu ya siku zote (More than half the days)
- ◯ Karibu kila siku (Nearly every day)

131) 9. Katika wiki mbili zilizopita, ni kwa siku ngapi umekuwa ukisumbuliwa na mawazo kuwa ni heri kufa au kujiumiza kwa njia nyingine?
(In the last two weeks, how often have you been bothered by thoughts that you would be better off dead or hurting yourself in some way?)

- ◯ Hakuna kabis (Not at all)
- ◯ Siku kadhaa (Some Days)
- ◯ Zaidi ya nusu ya siku zote (More than half the days)
- ◯ Karibu kila siku (Nearly every day)
