# Supplementary material for: A mixed-methods comparison of gender differences in alcohol consumption and drinking characteristics among patients in Moshi, Tanzania
Source: PLOS Glob Public Health. 2023 Oct 24;3(10):e0002009. doi: 10.1371/journal.pgph.0002009 (PMC10597514; doi:10.1371/journal.pgph.0002009)
Supplement: S2 Questionnaire — (PDF) [file pgph.0002009.s007.pdf]

Interview Guide (Gender Differences in Alcohol Use)

Time of interview/ ***Muda wa mahojiano:***

Date/ ***Tarehe***:

Place/ ***Mahali***:

Interviewer/ ***Mhojaji***:

Interviewee/ ***Mhojiwa***:

Hello, thank you for taking the time to speak with me. Today we will discuss your opinions and beliefs on alcohol. Our purpose in asking these questions is to understand if there are differences in women's and men's drinking behaviors and if so, to consider how we can improve treatment programs and reduce alcohol-related harm. I really appreciate that you are willing to take the time to share your views. Please stop me if a question does not make sense and you would like me to clarify it, if something makes you feel uncomfortable, or if you would prefer not to answer. Your input is extremely important; there are no 'right' or 'wrong' answers to these questions. We would like to know your opinions, feelings, and experiences on this subject as openly as you are able to share. As a reminder, your responses are confidential, and will not influence your care. You are welcome to withdraw from this study at any time. / *Habari, asante kwa kuchukua muda wako kuzungumza nami. Leo tutajadili kuhusu maoni na mtazamo wako juu ya matumizi ya pombe. Lengo letu ni kutambua tofauti katika matumizi ya pombe/kileo baina ya wanawake na wanaume katika jamii, ili kuweza kuboresha matibabu na msaada kwa madhara yatokanayo na matumizi mabaya ya pombe. Ninashukuru sana kuwa uko tayari kuchukua muda wako kutoa maoni yako. Maoni yako ni muhimu sana na yatatusaidia kuboresha programu za kupunguza madhara yanayohusiana na pombe nchini Tanzania. Kuwa huru wakati wowote kuniuliza swali au maelezo zaidi, na kama itatokea chochote cha kukufanya usijisikie huru unaweza kuchagua kutokujibu. Kwa kukumbusha, majibu yako ni ya siri na hayataathiri matibabu yako kwa njia yoyote hivyo kuwa huru kuelezea kwa kadri unavyoweza.*

"This is an interview by__________ *(name of interviewer)* on ____________ *(date)* with participant ______________*(subject ID)*."

1. I would like to start our conversation by asking you to describe your community's views on alcohol. /*Ningependa kuanza majadiliano yetu kwa kukuomba ueleze maoni ya jamii yako juu ya pombe*.

a. What are the positive ways alcohol has affected your community, if any? *Ni kwa namna gani pombe imegusa jamii yako kwa namna chanya,*
b. What are the negative ways alcohol has affected your community, if any? *Ni kwa namna gani pombe imeathiri jamii yako kwa namna hasi, au kuna ubaya wowote wa pombe katika jamii yako.*
c. When is it okay to drink alcohol? (*Ni muda gani sahihi kunywa pombe?)*
    i. who can drink alcohol? (*Nani anapaswa kunywa pombe/ kutumia kileo?)*
    ii. Who cannot drink alcohol? *(Nani hapaswi kunywa pombe/ kutumia kileo?*
d. Do you think the way you view alcohol is different than how most others in your community view alcohol? If yes, how so? *(Je unadhani mtazamo wako kuhusu matumizi ya pombe ni tofauti na watu wengine katika jamii yako)*
e. How do you think women *(in general)* view alcohol?/ Unafikiri wanawake (kwa ujumla) wanaichukuliaje pombe?
f. How do you think men *(in general)* view alcohol?/ *Unafikiri wanaume (kwa ujumla) wanaichukuliaje pombe?*
g. What expectations does your community hold about alcohol use?/ *Je! Jamii yako ina matarajio gani juu ya matumizi ya pombe*
    i. Do these expectations differ between men and women? Why or why not?/ *Je! Matarajio haya yanatofautiana kati ya wanaume na wanawake? Kwa nini au kwa nini hapana?*
    ii. *(If yes, probe:)* How do they differ?/ (Ikiwa ndio, dodosa :) yanatofautiana vipi?
h. Is there a relationship between depression and alcohol use? If yes, please explain.
    i. If you can think of any examples, please share them with me.
    ii. Is the burden of alcohol use and depression higher among either men or women? Why or why not?

2. Please describe alcohol use among men in Tanzania/ *Tafadhali elezea matumizi ya pombe kwa wanaume nchini Tanzania*
    a. Is there stigma around men who drink? If so, please describe this stigma. / *Je! Kuna unyanyapaa kwa wanaume wanao kunywa pombe? Ikiwa ni hivyo, tafadhali eleza unyanyapaa huo.*
    b. Can you think of any man who has experienced stigma related to alcohol before? If you can think of an example, please share this with me.

(*Unaweza kumkumbuka mtu yoyote ambae alipata kunyanyapaliwa katika jamii kwa sababu ya matumizi yake ya pombe?)*

c. Why do you think men drink alcohol?/ *Unafikiri ni kwanini wanaume wanakunywa pombe?*
    i. What would lead a man to start unhealthy drinking habits?/*Ni nini kinachoweza kusababisha mwanamume kuanza kunywa pombe kwa kupitiliza?*
d. When would a man drink alcohol?/ *Je! Ni wakati gani mwanaume anakunywa pombe?*
e. What kinds of alcohol do men prefer? (*Ni pombe aina gani wanaume hupendelea kutumia?)*
    i. Why do men do you think men prefer these kinds of alcohol? (*Ni kwanini wanaume hupendelea pombe za aina hiyo tajwa hapo juu?)*
f. Where do men drink alcohol?/ *Wanaume hunywa wapi pombe?*
    i. Are there any places where it is not okay for men to drink?/ *Je! Kuna maeneo yoyote ambayo sio sawa kwa wanaume kunywa pombe?*

3. Please describe alcohol use among women in Tanzania/ *Tafadhali eleza matumizi ya pombe kwa wanawake nchini Tanzania*
    a. Is there stigma around women who drink? If so, please describe this stigma./ J*e! Kuna unyanyapaa kwa wanawake wanao kunywa pombe? Ikiwa ni hivyo, tafadhali eleza unyanyapaa huo.*
        i. Is the stigma around women who drink different than men who drink? If yes, why? In what ways is it different? (*Je unyanyapaa kwa wanawake wanaotumia pombe hutofautiana na unyanyapaa kwa wanaume?)*
        ii. Can you think of any woman who has experienced stigma related to alcohol before? If you can think of an example, please share this with me.
    b. Why do you think women drink alcohol?/ *Unafikiri ni kwanini wanawake wanakunywa pombe?*
        i. What do you think would lead a woman to start unhealthy drinking habits?/*Je unafikiri ni nini kinachoweza kusababisha mwanamke kuanza kunywa pombe?*
    c. When would a woman drink alcohol?/ *Je! Ni wakati gani mwanamke anakunywa pombe?*

    d. What kinds of alcohol do women prefer? /*Wanawake hupendelea aina gani ya pombe?*
        i. Why do you think women prefer these kinds of alcohol? / *Kwanini wanawake hupendelea aina hiyo ya pombe tajwa hapo juu?*
    e. Where do women drink alcohol?/ *Wanawake hunywa wapi pombe?*
        i. Are there any places where it is not okay for women to drink?/ J*e! Kuna maeneo yoyote ambayo sio sawa kwa wanawake kunywa pombe?*
        ii. Are the places where it is okay for women to drink different than the places it is okay for men to drink? If so, why?
        Je, kuna maeneo yoyote ambayo mwanawake wanakunywa tofauti na wanaume?

4. How do you think alcohol use differs between men and women?/ *Je, Unafikiri matumizi ya pombe yanatofautiana vipi kati ya wanaume na wanawake?*
    a. What are the consequences of drinking alcohol? /*Je! Ni nini athari za kunywa pombe?*
        i. How do these consequences differ between men and women?/ *Je! athari hizi zinatofautianaje kati ya wanaume na wanawake?*
    b. How are women who drink alcohol viewed?/ *Je! Wanawake wanaokunywa pombe wanaonekanaje/wanachukuliwaje?*
        i. Does this differ from men? *(If yes, probe:)* How does it differ?/*Je! Hii inatofautiana na wanaume? (Ikiwa ndio, dodosa :) Inatofautianaje?*

5. I would now like to ask some questions about alcohol use and pregnancy. My first question is, how does your community view alcohol use during pregnancy? / *Je jamii yako ina mtazamo gani kuhusu matumizi ya pombe wakati wa ujauzito?*
    a. What have you been told about alcohol use during pregnancy? Who has told you these things? / *Umewahi kusikia taarifa gani kuhusu matumizi ya pombe na umezisikia wapi?*
        i. Is drinking while pregnant helpful/good? If so, why? In what ways is it helpful? / *Je matumizi ya pombe katika ujauzito yana faida zozote? Kama ndio kwann?/ ni kwa namna gani inasaidia?*
        ii. Do you think that drinking while pregnant can lead to any problems for the unborn baby? Or for the mother? If so, can you please explain? In what ways is it harmful? / *Je pombe inaweza kuleta madhara katika ujauzito (kwa mama na mtoto)? Na ni madhara gani?*

iii. Can you think of any women you know who have drunk while pregnant? Did anything happen to them? / *Unamkumbuka mjamzito yoyote katika jamii yako ambae alikua akitumia pombe katika ujauzito, na kama alipata madhara yoyote?*

b. Does women's drinking behavior change when they are pregnant? If so, how? / *Je matumizi ya pombe kwa wanawake hubadilika kwa namna yoyote ile wanapopata ujauzito?*

c. How common is drinking during pregnancy among the women that you know? / *Je kutumia pombe katika ujauzito ni jambo la kawaida katika wanawake unaowafahamu?*

d. How are women who drink while they are pregnant viewed? / *Wanawake wanaotumia pombe wakiwa wajawazito wanatazamwa vipi katika jamii?*

   i. How is this different than drinking while *not* pregnant? / *Je mtazamo huo hapo juu unatofautiana na pale mwanamke huyu akiwa sio mjamzito?*

We are coming to the end of our interview and I want to thank you so much again for your time and responses. As I said at the beginning of our interview, the information that we gather in this project we want to use to create and improve programs that can help people who have harmful drinking habits. So, for our last questions, I am now going to ask you about risky drinking. *Tunapokaribia mwisho wa maongezi yetu, ninapenda kukushukuru sana kwa muda wako na majibu yote ambayo yatasaidia sana kutengeneza mpango kazi kupambana na madhara ya matumizi yasiyo sahihi ya pombe katika jamii yetu. Na kipengele cha mwisho ningependa tuongelee kuhusu matumizi hatarishi ya pombe/kileo.*

6. How should people who have unhealthy drinking habits seek help? / *Ni njia gani ambayo wanaweza kupata msaada watu wenye unywaji wa pombe hatarishi au uliopitiliza?*
   a. Do you know someone who has received help for alcohol use? Can you describe what happened? / *Unamjua mtu yoyote ambae amewahi kupata msaada/matibabu juu ya unywaji wake hatarishi, na msaada ulikua kwa njia gani?*
   b. What barriers exist for those seeking treatment for alcohol use? What might prevent someone from seeking treatment?
7. What is risky drinking behavior? / *Nini maana ya unywaji hatarishi wa pombe?*

a. What are the main risks of drinking alcohol? / *Nini hatari kubwa unazozifahamu za kunywa pombe/ kutumia kileo?*
b. When is alcohol-related harm most likely to happen? / *Ni wakati gani haswa madhara yatokanayo na unywaji pombe yanaweza kutokea?*

8. To stop risky drinking behavior, what types of support are needed?/ *Ili kuacha tabia hatarishi za unywaji wa pombe, ni aina zipi za misaada zinahitajika?*
    a. What would help people quit or reduce risky drinking behavior?/ *Ni nini kitakachosaidia watu kuacha au kupunguza tabia hatarishi za unywaji wa pombe?*
    b. Does support need to be different for men vs for women? Why or why not?/ *Je! Msaada unatakiwa kuwa tofauti kwa wanaume ukilinganisha na kwa wanawake? Kwa nini au kwa nini hapana?*
        i. *(if yes, probe:)* How would alcohol-reduction support be different for men than for women?/ *(ikiwa ndio, dodosa:) Ni jinsi gani ambavyo msaada wa kupunguza pombe unaweza kuwa tofauti kwa wanaume ukilinganisha na kwa wanawake?*
        ii. If you could design a program for people in your community to help them with unhealthy drinking, what would it look like? *Kama utabuni mango wa kuisaidia jamii yako kuhusiana na unywaji mmbaya je utafananaje?*
    c. Do you think individually-focused support or socially-based support would be more effective to stop risky drinking behavior? Why or why not?/ *Je! Unafikiri msaada unaozingatia mtu mmoja mmoja au msaada wa pamoja utakuwa bora zaidi kuacha tabia hatarishi za unywaji wa pombe? Kwa nini au kwa nini hapana?*
        i. Would men prefer socially-based support or individual support? Why?/ *Je! Wanaume wangependelea msaada wa pamoja au msaada wa mtu mmoja mmoja? Kwa nini?*
        ii. Would women prefer socially-based support or individual support? Why?/ *Je! Wanawake wangependelea msaada wa pamoja au msaada wa mtu mmoja mmoja? Kwa nini?*
