# Supplementary material for: A mixed-methods comparison of gender differences in alcohol consumption and drinking characteristics among patients in Moshi, Tanzania
Source: PLOS Glob Public Health. 2023 Oct 24;3(10):e0002009. doi: 10.1371/journal.pgph.0002009 (PMC10597514; doi:10.1371/journal.pgph.0002009)
Supplement: Attachment — Submitted filename: A Mixed gender AUDIT review.docx [file pgph.0002009.s008.docx]

**A Mixed-Methods Comparison of Gender Differences in Alcohol Consumption and Drinking Characteristics among Patients in Moshi, Tanzania**

A very relevant study for the study setting. This study also has relevant clinical implication which also would vary across gender. Systemic illnesses commonly for men while mental health problems for women and need to be further studied in this setting for more appropriate management. The areas necessary to be addressed are listed below.

Line 98 This paragraph is a repetition of the previous paragraph. I was expecting the researcher discuss past literature that possibly explained the variation in alcohol consumption by gender, looking at studies within the study region or the country or even beyond.

Line 105 This paragraph is the justification for this study and it needs to be expanded explicitly.

Line 133 What does this phrase means? “Injuries have long been associated with excessive alcohol use”.

Line 154 What does this phrase means? “No women participants presented to both the ED and RHC”.

The Quantative result section was not coordinately written. I would suggest the researcher give an initial report from the ED for males and females, then go on to report the findings from the RHC.

Also, Figure 2 was not explained in any part of the results. Also what are the levels of significance of all the findings across this study? Report your p values.

Line 413 “In general, respondents reported that men had greater agency around their drinking, meaning greater ability and access to control their own drinking behaviors”. There is no such statement as having agency around drinking, use appropriate and comprehensive terms.

In the Qualitative result, if the participants were chosen purposely (Line 293-295) then the result for these categories variation in gender alcohol consumption and characteristics should be reported to reveal the gender differences across various socio-cultural and socio-economic population.

Line 688 “boon” or “boom”?

Report the Implication of this study and the limitations in a separate sub-headings from discussion.
