# Supplementary material for: A mixed-methods comparison of gender differences in alcohol consumption and drinking characteristics among patients in Moshi, Tanzania
Source: PLOS Glob Public Health. 2023 Oct 24;3(10):e0002009. doi: 10.1371/journal.pgph.0002009 (PMC10597514; doi:10.1371/journal.pgph.0002009)
Supplement: Attachment — Submitted filename: Response to Reviewers - Consumption.docx [file pgph.0002009.s009.docx]

August 16th, 2023

To Whom It May Concern:

We appreciate the helpful suggestions presented to us by our reviewers and thank them for the opportunity to improve our manuscript. We have incorporated the changes requested by all reviewers in our attached submission, with our specific modifications outlined below. Our revised manuscript is attached with this resubmission packet.

Reviewer #1:

The manuscript examines the prevalence and patterns of alcohol use and alcohol use disorders in a select population from Tanzania. It is a mixed methods study with a cross-sectional survey and a nested qualitative study. While the study is robust, there are several gaps in reporting which will need to be addressed before the paper could be considered for publishing.

ABSTRACT
**Comment**: (Methods) Add a sentence on how both sets of data were analysed
*Response: We thank the reviewer for this suggestion. We have added more information on our analysis approach in the abstract. This addition reads, ‘Quantitative data was analyzed in RStudio through descriptive frequencies, proportions, ANOVA, and Chi-squared tests, while IDIs were analyzed in Nvivo following a grounded theory approach.’*

**Comment**: (Results) '.....alcohol use behaviors including lower rates of consumption among women...' AUDIT scores do not indicate 'rates' of consumption
*Response: We appreciate this feedback and have modified this sentence accordingly. In line with the below feedback, it now reads ‘...significant differences in their alcohol use behaviors. For quantitative data, this included lower average AUDIT scores…”*

**Comment**: (Results) Please separate the quant and qual results. At the moment you report both in a single sentence and it will confuse the reader
*Response: My co-authors and I thank the reviewer for this suggestion; the results section of the abstract has been modified accordingly.*

MAIN PAPER
**Comment**: Please check the manuscript for English and grammar. Examples of errors include 'While women intake less alcohol.....'; 'The data that does exist has indicated that while intoxicated, men are more likely to exhibit violent behaviors while women are more likely to be victims of violence.' ‘Standing also as contributing factors is alcohol’s ready availability….’
*Response: We apologize for these errors. We have fixed these mistakes and have performed a comprehensive English and grammar review of our entire manuscript. All identified mistakes have since been corrected.*

INTRODUCTION
**Comment**: The 'aim' is very broad. Please supplement with specific objectives
*Response: We thank the reviewer for this suggestion. We have further specified the aim of this paper: “...this study aims to better understand key gender differences in alcohol use among patients in Moshi, Tanzania, by (1) quantitatively comparing measures of consumption and the relative risks of alcohol misuse among the patient populations studied, and (2) qualitatively exploring the distinctions between men and women’s alcohol use behaviors.” This can be found at the end of the introduction section.*

METHODS
**Comment**: Is there a potential for selection bias- those with injuries are more likely to be consuming alcohol heavily? Also, did you exclude pregnant women attending the gynecology clinic? If not, would that also be a sample with potential selection bias i.e. pregnant women might be more likely to reduce/stop drinking
*Response: We thank the reviewer for these questions. To answer your initial inquiries, the ED was chosen as a clinical site as we anticipated that this population would indeed be consuming alcohol more heavily than the general population, and pregnant women were not excluded from this study. To better clarify this point, we have added, ‘Pregnancy was not an exclusion criterion for this study’ within the section “Methods: Participants.” Both of these patient groups were purposefully sampled to help us better understand the varying drinking patterns among patients in this region.*

*Beyond comparing alcohol use between men and women, our paper sought to assess which patient population had the highest risk of alcohol misuse and might be most amenable to alcohol-reduction-related interventions to reduce it. We thus hold the variables of injury or pregnancy as risk factors, rather than selection biases, intentionally studied to help us better understand consumption patterns and risk. To improve our manuscript and clarify this important point, we have better specified this paper’s aim, in line with the suggestion above.*

**Comment**: 'No women participants presented to both the ED and RHC'. How would you know if they presented at these two settings at two different time points during the course of recruitment?
*Response: We appreciate this question from the reviewer. During the first half of the study, each research assistant was assigned to enroll patients from a single clinical unit and so was able to recognize any individuals who had previously participated. When our two female research assistants switched enrollment locations, while explaining the study, they asked patients verbally if they had participated in the past. If answering affirmatively, these patients were not enrolled. The main data analyst confirmed this through a data search using patient initials and age. We have additionally clarified this sentence to read, ‘At the time of enrollment, patients verified that they had not previously participated in this study either in the ED (male and female patients) or the RHC (female patients). This ensured each participant was only enrolled once.’*

**Comment**: 'As surveys were collected at a single time point, there were no patients lost to follow-up' This sentence is superfluous
*Response: We thank the reviewer for this feedback. This sentence has been removed.*

**Comment**: 'Quantitative surveys consisted of five main components: (1) basic demographic data, (2) self-reported alcohol use data......' Was #2 just a binary yes-no question or a set of questions? If it was the later, then please provide details
*Response: We appreciate this question. In line with your suggestion, we have elaborated on this sentence. It now reads, ‘...(1) basic demographic data, (2) self-reported alcohol use data consisting of scaled multiple choice or binary yes-no questions…’. Additionally, we have uploaded our full survey questions template along with our interview guide as supporting appendices.*

**Comment**: What do you mean by the 'depression module' of PHQ-9. The whole PHQ-9 is a tool for identifying potential depression
*Response: We thank the reviewer for this clarification and acknowledge our unclear wording. We have updated this sentence to now read ‘...the nine-item Patient Health Questionnaire (PHQ-9).’*

**Comment**: If you are not reporting PHQ-9 and DrInC in this manuscript then please do not describe in methods section. Suggest reporting at least mean PHQ-9 and DrInC scores for each one of the groups
*Response: We thank the reviewer for bringing this incongruity to our attention. We have removed the description of these tools from the methods section as we do not plan to report these scores in this paper. It is in other depression and consequence-specific manuscripts (currently under production) that we plan to report average PHQ-9 and DrInC scores.*

**Comment**: Please move the following to the section related to data and not in the analyses: 'Consumption was also measured through self-reported alcohol consumption questions that asked participants how much and how often they consumed alcohol, what types of alcohol they preferred, and how much money they typically spent on alcohol per week.'
*Response: We thank the reviewer for this suggestion. We have moved this sentence to “Methods: Quantitative Data: Instruments.”*

**Comment**: Line #279 There are no sample size calculations in qual studies. Please label this section as 'sample' and merge the content from 'procedures' below as it describes how you tried to achieve maximum variability in the sample on various parameters
*Response: We appreciate this comment and have made the suggested edit.*

**Comment**: How was consent recorded in illiterate participants? Was any care provided to those who screened positive on the AUDIT?
*Response: We thank the reviewer for these questions and for the opportunity to strengthen our manuscript in answering them. For illiterate patients, consent was recorded via their initials if able, and if not, through a cross mark on their consent form. Patients who screened positively for unhealthy alcohol use via the AUDIT scale were referred to the for further care at KCMC. This has been added to the manuscript under “Methods: Quantitative Data: Procedures” and “Methods: Quantitative Data: Instruments,” respectively.*

RESULTS
**Comment**: Tables 2 and 3: Since the denominator remains the same for each block, please do not report it for each cell in that block. Just report the missing value at the bottom of each block.
*Response: We thank the reviewer for this suggestion and have altered tables 2 and 3 as such. We have also removed the total missing/refused from the variable heading, as this recent addition made this detail repetitive.*

**Comment**: Table 1: Please report mean age (SD) as well.

*Response: We thank the reviewer for this suggestion. We have added statistics on the mean and standard deviation for patients’ ages.*

**Comment**: Why do you need to report both personal and household income? Please report just one i.e. the one which is most representative of SE status in this setting.
*Response: We thank this reviewer for this suggestion and have removed the variable ‘household income category’ from this report.*

**Comment**: Please arrange the categories for all the variables in Tables 1 and 2 in a logical sequence e.g. None -> Primary --> Secondary etc
*Response: We thank the reviewer for this suggestion and have rearranged these tables as such.*

**Comment**: Table 3: I am not sure how you have merged drinking frequency (times/week) with intensity (multiple times a day). For example, the person drinking 1-2 times per week could be drinking multiple times on those days
*Response: We appreciate this question from the reviewer. These values have not been merged. Rather, we asked patients separately how many times per week they drink and how many standard drinks they consume per sitting. We have clarified these variable names in Table 3 and have also provided a definition of standard drinks under our section, “Methods: Quantitative Data: Instruments.” This addition reads, ‘Participants were asked to report their alcohol consumption in terms of standard drinks, with one standard drink being defined as any beverage containing 10 g of pure alcohol, in line with WHO guidelines.’*

**Comment**: Table 3: Quantity over what period/drinking session? Also, are all the drinks consumed as bottles? I would assume not. Especially the 'spirits'
*Response: We thank the reviewer for this question and acknowledge that this had been unclear as previously written. Respondents were asked to report drinking quantity in terms of standard drinks. Further, this intake was measured per sitting. This has been updated as such in Table 3.*

**Comment**: Please also report mean (SD) and range for expenditure on alcohol
*Response: We thank the reviewer for this suggestion. Unfortunately, we collected alcohol expenditure exclusively as a categorical variable (How much do you spend on alcohol (per week)? (a) 0 to 10000 TZS, (b) 10000 to 50000 TZS, (c) 50000 TZS to 100000 TZS, (d) >100000 TZS, or (e) Refused/Don’t Know) and thus are unable to report the mean, SD, or range for alcohol expenditure.*

**Comment**: Please arrange logically (No followed by yes). Please ensure that you check all the variable and ensure that there is a logical reporting of the categories in each variable. Why is 'refused/do not know' not excluded as missing data for all these variables? Not doing so is leading to an inaccurate synthesis of data
*Response: We thank the reviewer for this comment and have combined the values for ‘refused/do not know’ with our missing values. We have also re-checked the categories for each of our quantitative variables to ensure logical reporting.*

**Comment**: I do not think figures 2 and 3 are adding anything to the narrative and can be excluded
*Response: We appreciate this suggestion. We have removed Figures 2 and 3 from this manuscript.*

**Comment**: IDI number is not required. For each quote please report, gender, age, and mean AUDIT score
*Response: We thank the reader for this suggestion. We have added gender, age, and AUDIT score for each IDI participant. While we understand that an IDI number is not required, to showcase the variety of participants represented in our qualitative section, we have kept IDI numbers. As an example, the new format for our first IDI is written as IDI #1, 24F, AUDIT = 0 after their respective quotes.*

**Comment**: Line #743 use appropriate terms. You cannot measure incidence through a survey
*Response: We thank the reader for bringing this mistake to our attention. We have rephrased the sentence to ‘...we found that alcohol misuse was highest within the ED at KCMC.’*

DISCUSSION
**Comment**: The discussion is focused around the qual findings and some about the prevalence in the survey. What about all the other details related to prevalence of drinking etc. None of that has either been discussed or triangulated with the qual findings

*Response: We appreciate this comment from the reviewer and agree that the quantitative and qualitative data should ideally be triangulated. However, within the constraints of the data collected from this study, my authorship team and I are limited in our ability to do so. Our findings of the greatest significance for alcohol-related harm reduction and future intervention planning were (1) the social implications of alcohol use for men and women and (2) the differing amounts of consumption among our patient populations. While the latter point is supported by both qualitative and quantitative data, the first point lacks a quantitative basis as survey questions related to the social implications of alcohol use were not asked. We acknowledge this gap in our results and thus have added it to our limitations section. This addition now reads, ‘...the study from which this data draws lacks quantitative data on the social implications of alcohol use, which limits our ability to triangulate some of our qualitative findings. Future studies should thus aim to address this gap to ensure the validity of these results.’*

Reviewer #2

A very relevant study for the study setting. This study also has relevant clinical implications which also would vary across gender. Systemic illnesses commonly for men while mental health problems for women and need to be further studied in this setting for more appropriate management. The areas necessary to be addressed are listed below.

**Comment**: Line 98 This paragraph is a repetition of the previous paragraph. I was expecting the researcher discuss past literature that possibly explained the variation in alcohol consumption by gender, looking at studies within the study region or the country or even beyond.
*Response: We appreciate this comment from the reviewer and have added more information explaining the variation in alcohol use by gender. We have also better differentiated this paragraph from the one proceeding it to explore gender differences on a global versus a local scale, respectively. However, as noted in the first sentence of this paragraph, data on why alcohol use differs is close to nonexistent in this region, not only limiting our ability to explore this topic in-depth but also underlying our motivation for putting forth this manuscript.*

**Comment**: Line 105 This paragraph is the justification for this study and it needs to be expanded explicitly.
*Response: We thank the reader for bringing this gap to our attention. We have expanded the justification for the study at the beginning of this paragraph, as well as further specified the aim of the study at the end of the paragraph.*

**Comment**: Line 133 What does this phrase means? “Injuries have long been associated with excessive alcohol use”.
*Response: We thank the reviewer for this clarifying question and acknowledge our unclear wording. We have rephrased this sentence to now read, “The intoxicating effects of alcohol have long been implicated in the onset of serious injuries, such as road crashes or violent assault, which necessitate emergency care. As a result, EDs see a higher proportion of patients reporting alcohol misuse than is typically found in the general population.”*

**Comment**: Line 154 What does this phrase means? “No women participants presented to both the ED and RHC.”
*Response: We thank the reviewer for this clarification. We have revised this sentence to now read, ‘At the time of enrollment, patients verified that they had not previously participated in this study either in the ED (male and female patients) or the RHC (female patients). This ensured each participant was only enrolled once.’*

**Comment**: The Quantative result section was not coordinately written. I would suggest the researcher give an initial report from the ED for males and females, then go on to report the findings from the RHC.
*Response: We thank the reviewer for this suggestion. We chose to concurrently report our data on ED males, ED females, and RHC females so as to highlight the differences in alcohol consumption between the two female populations alongside deviations in gender, as we believe that this will help guide future intervention planning. Beyond this, an explanation of ED patients that is separate from RHC patients may create unnecessary repetitions in our results section that may be confusing for the reader. While we respect and see the merit in this suggestion from the reviewer, we believe that the results as written illustrate our data most effectively while also eliminating any unnecessary back-and-forth.*

**Comment**: Also, Figure 2 was not explained in any part of the results.

*Response: We acknowledge this gap in our results and thank the reviewer for bringing this to our attention. In considering this point, we have removed Figure 2 from the manuscript as it does not add new information to our overall narrative.*

**Comment**: Also what are the levels of significance of all the findings across this study? Report your p values.
*Response: We thank the reviewer for this feedback. We had added P-values across all reported variables. We have also expanded our section, “Methods: Quantitative Data: Analysis,” to describe the methods behind our siginficane testing.*

**Comment**: Line 413 “In general, respondents reported that men had greater agency around their drinking, meaning greater ability and access to control their own drinking behaviors”. There is no such statement as having agency around drinking, use appropriate and comprehensive terms.
*Response: We thank the reader for this feedback. We have replaced the word ‘agency’ in this sentence with ‘control.’*

**Comment**: In the Qualitative result, if the participants were chosen purposely (Line 293-295) then the result for these categories variation in gender alcohol consumption and characteristics should be reported to reveal the gender differences across various socio-cultural and socio-economic populations.
*Response: We thank the reviewer for this suggestion. We had omitted a more detailed overview of our in-depth interviewee demographics as this exists in a separate manuscript. Having also used data from this study, our manuscript, “Understanding the Impacts and Perceptions of Alcohol Use in Northern Tanzania: A Mixed-Methods Analysis,” which is currently under review with Plos GH, contains a table dedicated to reporting these qualitative characteristics. To ensure that this paper contains entirely original, previously unpublished work, we had intended to abstain from incorporating a similar table here.*

**Comment**: Line 688 “boon” or “boom”?
*Response: We intended the use of the word ‘boon’ as written. In this sentence, we use ‘boon,’ meaning ‘a thing that is helpful or beneficial,’ in the context of alcohol’s impact on men’s social status.*

**Comment**: Report the Implication of this study and the limitations in separate sub-headings from discussion.

*Response: We thank the reader for this feedback and have added a separate sub-heading for the limitations. Additionally, we have expounded upon our study implications at the beginning of this section.*
